# A histochemical study of the Nras/let-60 activity in filarial nematodes

**DOI:** 10.1186/s13071-015-0947-6

**Published:** 2015-07-01

**Authors:** James F. Geary, Raquel Lovato, Samuel Wanji, Ron Guderian, Maeghan O’Neill, Sabine Specht, Nicole Madrill, Timothy G. Geary, Charles D. Mackenzie

**Affiliations:** Department of Pathobiology and Diagnostic Investigation, Michigan State University, East Lansing, MI 48824 USA; Ecuadorian Onchocerciasis Control Program, Ministry of Health, Quito, Ecuador; Research Foundation for Tropical Diseases and Environment, P.O. Box, 474, Buea, Cameroon; Hospital Vozandes, Quito, Ecuador; Institute of Parasitology, Macdonald Campus, McGill University, Ste-Anne-de-Bellevue, QC H9X 3V9 Canada; Institute for Medical Microbiology, University Hospital Bonn, Bonn, Germany; Liverpool School of Tropical Medicine, Pembroke Place, Liverpool, L35QA UK

**Keywords:** *Onchocerca*, Nras, let-60, *Wolbachia*, Mutualism, Filariae

## Abstract

**Background:**

Control and elimination of filarial pathogens is a central focus of major global health efforts directed at parasitic diseases of developing countries. Accomplishment of these goals would be markedly enhanced by the enhanced destruction of the adult stage of filariae. The identification of new, more quantitative biomarkers that correlate with mortality or chemotherapeutic damage to adult filariae, would greatly facilitate, for example, the development of new macrofilaricides.

**Methods:**

An immunocytochemical approach using an antibody against human Nras was used to identify and detect changes in the nematode homolog let-60 that is associated with cell growth and maintenance. Single *Onchocerca volvulus* nodules were removed from each of 13 patients treated with ivermectin (as part of a community-wide mass drug administration programme), and from each of 13 untreated individuals; these 26 nodules were stained with the anti-Nras antibody. The localization and degree of positivity of Nras/let-60 staining were assessed subjectively and compared between the two groups; the positivity of staining was also quantified, using image analysis, in a subgroup of these nodules. In addition, the specific morphological association between Nras/let-60 and the *Wolbachia* endosymbiont present in these parasites was also observed in 4 additional filarial species using an anti-*Wolbachia* surface protein (WSP) antibody under light and confocal microscopy.

**Results:**

Nras/let-60 is present in many structures within the adult female worms. A statistically significant decrease in the general staining intensity of Nras/let-60 was observed in adult female *O. volvulus* treated with ivermectin when compared with parasites from untreated patients. Nras/let-60 staining was frequently observed to be co-localized with WSP in *O.volvulus, Brugia malayi*, *Litomosoides sigmodontis* and *Dirofilaria immitis*. Nras/let60 is also present in *Onchocerca ochengi*.

**Conclusion:**

Nras/let-60, as detected by immunocytochemical staining, is decreased in ivermectin-treated adult female *O. volvulus* relative to untreated control specimens, suggesting a suppressive effect of ivermectin on the overall biochemical activity of these parasites. Co-localization of Nras/let-60 and WSP suggests the possibility that the endosymbiont utilizes this nematode protein as part of a mutualistic relationship. Nras/let60 appears to be a useful biomarker for assessing the health of filariae.

**Electronic supplementary material:**

The online version of this article (doi:10.1186/s13071-015-0947-6) contains supplementary material, which is available to authorized users.

## Background

Filarial parasites cause some of the most debilitating and chronic diseases of humans. Two of these, onchocerciasis and lymphatic filariasis, are targeted for control and elimination largely through chemotherapeutic approaches. *O. volvulus,* a filarial nematode belonging to the superfamily Filarioidea transmitted through an arthropod vector of the *Simulium* genus, causes both dermal and ocular pathology. An estimated 120 million people remain at risk with over 300,000 having been blinded by the disease. Approaches that target the adult filaria would likely enhance the current global health goals of controlling and eliminating these diseases but this has proven to be elusive although remains the subject of much current research [1–4].

Current drug therapy of onchocerciasis relies primarily on eliminating transmission by interrupting the life cycle by reducing dermal microfilarial loads using ivermectin to kill these infectious parasites [5] and to block their release from the uterus of adult female worms. This drug opens glutamate-gated chloride channels, paralyzing nematode neuromuscular systems [6]. In filariae, this action may be most important in inhibition of the release of excretory-secretory products [7] and suppression of the release of microfilariae (mff), perhaps by inhibition of feeding or ovijector function. However, ivermectin does not cause rapid death of adult parasites, although it is thought to shorten the life span to some extent [8]. A better understanding of the effect of ivermectin on the adult worms would be most useful.

Like most filariae, *O. volvulus* contains the endobacterial obligate intracellular mutualist *Wolbachia* [9]. *Wolbachia* are alpha-proteobacterial entities that have lost many of the biological pathways needed to survive outside a carrier host [9, 10]. Because *Wolbachia* are necessary for the long-term survival of adult *O. volvulus*, the bacterium is a target for chemotherapy with agents such as doxycycline, which has been shown to slowly kill adult *O. volvulus* [11, 12]. *Wolbachia* are transmitted vertically, being introduced to the oogonia from the uterine wall [13, 14], and are thought to contribute to several metabolic pathways that aid in the survival of the worm; these include the production of ribonucleotide precursors and of heme. The production of this important iron-containing electron transfer agent depends on *Wolbachia*, since filariae that harbor them are incapable of producing heme themselves [10]. Again a more comprehensive understanding of the biology of the interaction between *Wolbachia* and *Onchocerca* species is likely to be useful in the search for novel macrofilaricides.

Ras proteins (small GTPases) play important roles in cellular signal transduction pathways in eukaryotic cells. They are trafficked throughout the cell and serve in rapidly switching systems for activating multiple processes in the cell, including those essential for cell survival, differentiation and growth [15]. The three ras proteins of humans (H- K- and N-) are all homologous to the let-60 protein in *Caenorhabditis elegans,* which is highly conserved in *Brugia malayi*, *Loa loa* and *O. volvulus. C. elegans* let-60 is involved in multiple processes, including excretory tube and vulval development [16]. Mutations in let-60 impact survival and development in *C. elegans*, as is the case in mammals [17]. In mammals, the three ras proteins show a high degree of sequence homology despite being encoded by three different genes; this homology does not confer redundant activity. Ras proteins localize primarily to the plasma membrane but also associate with mitochondria and the nucleus [18, 19]. In *C. elegans,* let-60 localizes to a variety of tissues [16], and has been reported to contribute to germ line morphogenesis in this nematode [20].

A challenge to antifilarial discovery and epidemiological monitoring of onchocerciasis in control programs is the dearth of validated methods to characterize the viability of worms recovered from hosts [1–4]. Based on the availability of cross-reactive antibodies and the putatively essential roles of proteins involved in signaling pathways, we evaluated mammalian immunocytochemical reagents against AKT-1, WNT-2b and Nras for their staining abundance and pattern in treated vs. untreated specimens of *O. volvulus*. The most promising of the antibodies tested was one raised against Nras, and we report here the results of a morphological approach to localize Nras/let-60 staining in filarial nematodes and compare ivermectin-treated and untreated adult female *O. volvulus*. In addition, because of the requirement of *Wolbachia* for the maintenance of adult *O. volvulus*, we also examined the relationship between Nras/let-60 and *Wolbachia* in various life cycle stages of *O. volvulus* and in other filariae.

## Methods

### Ethics statement

The *O. volvulus* nodules used in this study were obtained as part of treatments carried out by the programmes of the National Onchocerciasis Control Program of Ecuador and at the University of Buea, Cameroon, using standard sterile surgical procedures approved by the appropriate local regulatory authorities. The mass drug administration programme in Ecuador was approved by the Ministry of Health. *B. malayi* and *L. sigmodontis* parasites were obtained from gerbil (*Meriones unguiculatus*) infections approved by the laboratory animal use committees of Michigan State University, McGill University and the Medical University Hospital, Bonn; all animals were housed in approved university facilities and maintained under approved ethical principles for animal experimentation and use. *D. immitis* specimens were obtained at necropsy from dogs euthanized by animal control authorities in Grand Cayman under the oversight of the Veterinary School of St. Mathews University. *O. ochengi* material was obtained from Kumba abattoir in Cameroon as part of the routine activity of these establishments. Nodules from doxycyline treated patients were provided from a study carried out in Ghana [21].

### Parasites

Untreated *O. volvulus* nodules were collected as part of pre-ivermectin nodulectomy campaigns in Ecuador or Cameroon from individuals who were known not to have received any anti-filarial treatment. Treated nodules were collected as an activity of the Onchocerciasis Elimination Program in Ecuador, which utilized a once or twice a year distribution of ivermectin. Adult *B. malayi* were isolated from the peritoneal cavity of gerbils infected by injection of L3s isolated from infected mosquitoes; these parasites were isolated from the peritoneal cavity upon necropsy. Adult *D. immitis* were collected at necropsy from dogs (*Canis lupus familiaris*). Adult *L. sigmodontis* worms were isolated from the pleural cavity of gerbils infected naturally by introducing infected mites into cage bedding. *C. elegans* were prepared in standard culture systems and were collected and maintained in phosphate-buffered saline (PBS) using standard procedures [22]. *O. ochengi* were dissected from the nodules in cattle hides collected from the local abattoir. All worms were fixed in 3.8 % buffered formaldehyde (10 % formol) for at least 48 h followed by storage in 60 % ethanol before routine preparation for paraffin embedding and sectioning.

### Immunocytochemical reagents

A BlastP search of the *B. malayi* proteome revealed the presence of a homolog of let-60 [GenBank: XP_001899045.1], which has a high degree of sequence homology with human Nras [GenBank: NP_002515], indicating a high likelihood that an antibody raised against human Nras would recognize the nematode protein (http://www.ebi.ac.uk/Tools/psa/emboss_needle/). A second BlastP search identified let-60 in the *O. volvulus* genome available through the Sanger Institute (http://www.sanger.ac.uk/resources/downloads/helminths/onchocerca-volvulus.html). An additional BlastP search demonstrated that a let-60 homolog is also present in the genome of *L. loa* [Genbank XP_003139513.1], a filarial nematode known to be free of *Wolbachia* [23]. Sequence comparisons are presented in Additional file 1: Figure S1.

To confirm that the polyclonal anti-human Nras antibody used in this study (Lifespan Biosciences, lot # 16276, Cat: LS-B2501) specifically recognizes nematode let-60, a Western blot experiment was run to determine the binding pattern of this antibody to proteins in a *C. elegans* extract in a standard protocol [22]. Briefly, 20 μg of *C. elegans* extract was electrophoresed through a 10 % polyacrylamide gel at 200 V. Transfer of proteins to a nitrocellulose membrane was performed at 400 mA. The primary antibody was incubated overnight with the membrane at 4 °C. The secondary antibody was added and incubated at room temperature for 1 h. Finally, 1 mL of 1:1 ECL reagent (Bio-Rad Hercules, CA) was added and the gel was developed. The resulting gel showed a strong band at 20–21 kDa (not shown), which is in agreement with the predicted molecular mass of the *C. elegans* let-60 protein at 21 kDa.

### Tissue and worm samples

Nodule and worm specimens were processed, embedded in paraffin and sectioned on a rotary microtome at 4 μm. Sections were placed on slides coated with 3-aminopropyltriethoxysilane and dried at 56 °C overnight. The slides were subsequently deparaffinized in xylene and hydrated through descending grades of ethyl alcohol to distilled water. Slides were then placed in Tris-buffered saline (pH 7.5; TBS) for 5 min for pH adjustment in preparation for the specific staining procedures. Twenty-six nodules, each from a separate patient, were examined in this study, i.e. 13 from treated people and 13 from untreated cases; these were randomly selected from a much larger archive group of ivermectin-treated and untreated individuals. Following investigation of these 26 nodules, 3 nodules from each treatment group were selected at random for a more detailed study where 5 worm sections from each nodule were selected for quantitative image analysis. This number was validated by a population sample size calculation performed with preliminary data. The Ecuadorian nodules analyzed were ivermectin-treated whilst the Cameroonian nodules acted as untreated controls.

### Immunocytochemistry

#### Nras staining

Sections were heat treated using a vegetable steamer (100 °C) for 30 min in a pH 6.0 citrate solution. Endogenous peroxidase was blocked using a 3 % hydrogen peroxide/methanol bath for 20 min followed by a running tap and distilled water rinses. Slides were then placed in TBS + Tween 20 and stained with an avidin/biotin complex (Vector Laboratories, Burlingame, CA). These staining steps were performed at room temperature on a DAKO Autostainer (Dako, Carpinteria, CA). After blocking non-specific staining with normal horse serum (Vector Labs) for 30 min, sections were incubated with an avidin (Vector labs)/biotin (Sigma-Aldrich, St. Louis, MO) blocking system for 15 min. Following subsequent rinsing in TBS + Tween 20, the slides were incubated for 30 min with a polyclonal goat antibody against Nras (Lifespan Biosciences, lot # 16276, Cat: LS-B2501) which was diluted 1:100 with normal antibody diluent (NAD) (Scytek, Logan, UT). Slides were then rinsed in two changes of TBS + Tween 20. After rinsing, the slides were incubated in biotinylated horse and goat IgG H + L (Vector Labs) in NAD at 11 μg/ml for 30 min. Slides were rinsed in TBS + Tween 20 and then the RTU Vectastain Elite ABC reagent (Vector Labs) was applied for 30 min. These slides were then rinsed with TBS + Tween 20 and developed using Nova Red (Vector Labs) for 15 min. At the completion of these steps, the slides were rinsed in distilled water, counter-stained with Gill 2 Hematoxylin (Thermo Fisher, Waltham, MA) for 30 s, differentiated in 1 % glacial acetic acid and rinsed in running tap water. Slides were then dehydrated through ascending grades of ethyl alcohol; cleared through several changes of xylene and cover-slipped using Flotex permanent mounting media (Lerner, Pittsburgh, PA).

#### WSP staining

Similar procedures were used to prepare WSP stained sections, with the exception that the heat-retrieval step was not applied. The mouse monoclonal anti-WSP (IgG) was obtained from BEI Resources (Cat: NR-31029, ATCC, Manassas, VA). Secondary antisera were biotinylated horse anti-mouse antibodies (Jackson ImmunoResearch, West Grove, PA) at 11 μg/ml (1:250 dilution). These slides were processed for Nova Red chromagen development and counterstaining as for Nras/Let-60. In this study, serial sections were always used: one stained with anti-Nras and the other with anti-WSP.

#### Assessment of staining

The extent and intensity of staining was assessed initially using a semi-quantitative subjective approach comparing parasite staining with host cell positive staining (plasma cells) in the same section with that occurring in the parasite’s components. A grading score of 0–3 was used (Table 1) for assessing the uniformity of distribution of the stain positivity in a particular anatomical structure. - 0 = no staining present, 1 = limited presence, 2 = moderately present, 3 = predominantly present and NP = stage not present in worm. The intensity, or strength, of staining present relative to that in the control human cell present in the same samples was also scored semi-quantitatively using a four stage system: 0 = no staining; 1 = weak staining; 2 = moderate staining; 3 = strong staining. A smaller group of three nodules from both treated and untreated samples were examined quantitatively using image analysis.

**Table 1:** Distribution and relative staining intensity for Nras/let-60 in untreated and ivermectin-treated *O. volvulus* adult female worms

Anatomical location	Untreated parasites		Ivermectin treated parasites	
	Uniformity^a^	Intensity^b^	Uniformity^a^	Intensity^b^
Hypodermis	3	3	2	1
Uterine epithelium	3	3	1	1
Intestinal epithelium	2	2	2	1
Oocytes	3	3	3	1
Morulae	3	3	NP	NP
Coiled mff	3	3	NP	NP
Mature mff	3	3	NP	NP
Spermatocytes	3	3	2	1
Spermatozoa	1	1	1	1

^a^Uniformity score: Based on the overall distribution of the staining throughout the particular anatomical structure. Range: 3 = predominantly present; 2 = moderately present; 1 = limited presence; 0 = no staining present, NP = stage not present in worm

^b^Intensity Score: Based on the overall intensity observed in all positive locations compared to human tissue control in the same section. Range: 0 = no staining; 1 = weak staining; 2 = moderate staining; 3 = strong staining

A nodule section contained between 12 and 25 individual sections of adult worms. Whereas the hypodermis was present in all these worm slices, and was assessed in all sections, many of the other areas of interest (e.g. morulae, germinal areas) were present in fewer of the worm sections present in each nodule and therefore were observed less frequently. The subjective score of a particular nodule was obtained by assessing all of the worm sections present in that nodule.

### Co-localization studies

Sections prepared for confocal microscopy followed the same procedures for each antibody as described above. The fluorescently labeled second stage antibody reagents used with the two primary antibodies, both diluted to 1:500, were a donkey anti- mouse IgG labeled with Alexa Fluor® 647 (Life Technologies, Grand Island, NY ) for WSP (red) and a donkey anti- goat IgG labeled with Alexa Fluor® 488 (Life Technologies) for Nras (green). Each antibody was applied for 30 min before rinsing.

### Image analysis

**Fig. 1:**
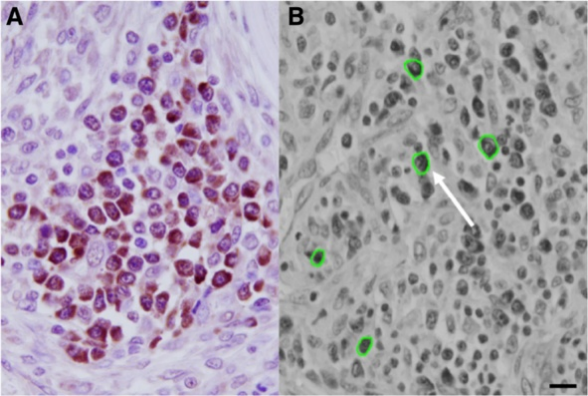
**a** Anti-Nras staining of host cellular reaction associated with adult *O. volvulus* demonstrating the intensity of Nras staining in the plasma cells and the general absence of staining in other host cells. **b** 8-bit grayscale converted image of host plasma cell zone, showing the selection of positive cells (arrow) as control values for quantification purposes

The computer programme ImagePro® (Media Cybernetics, Rockville, MD) was used to perform the image analysis. An image of each nodule was taken using an Olympus DP71 microscope mounted camera (Olympus, Center Valley, PA) captured at the highest resolution available and maintained in .*tiff* format to avoid degradation through file compression. Host plasma cells were selected by visual comparison to establish the most densely stained entities to act as the internal controls. No statistically significant difference was observed between the plasma cell controls of ivermectin-treated and the untreated nodules; this concurred with the subjective visual assessment; 5 cells was determined to be statistically a sufficient number to count (*p* > 0.05). Cameroonian tissues from untreated people were considered to be appropriate controls for the treated tissues from Ecuador as a comparison between these two groups, assessing for similarity in staining intensity of the standard control component (host plasma cells), showed no obvious difference in the staining of these cells between these two groups. The images of the plasma cells were converted to gray scale format using ImagePro®, permitting a bitmap analysis to be performed (Fig. 1). A bitmap is expressed in gray scale units, 0 being black and 255 being white; this form of analysis enables an easy comparison between different images by maintaining a constant scale and the ability to analyse multiple sections from multiple nodules without being biased by colour.

**Fig. 2:**
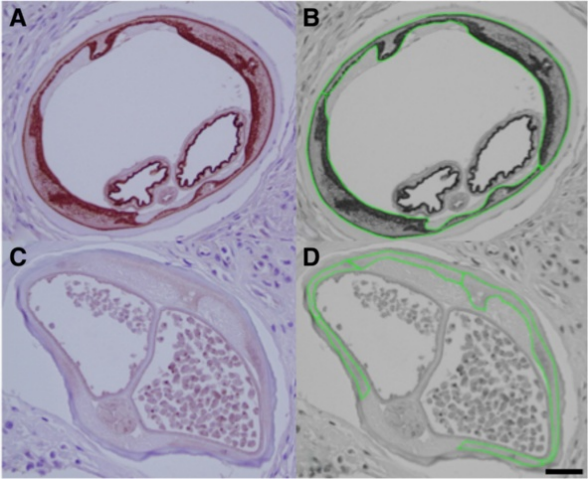
**a** Anti-Nras/let-60 stained section of an untreated adult female *O. volvulus* demonstrating the staining pattern most commonly observed with staining in the hypodermis and uterine epithelium. **b** 8-bit grayscale converted image of Fig. 4a demonstrating the selection of areas for analysis. **c** An anti-Nras/let-60 stained section of an ivermectin-treated adult female *O. volvulus* demonstrating the decrease in staining pattern most commonly seen in ivermectin-treated nodules. **d** 8-bit grayscale converted image of Fig. 4c demonstrating the selection of the area for analysis

In the same manner, 5 worm sections were selected around the geometric centre of the nodule and these images were also converted to gray scale for bitmap analysis (Fig. 2).

The analysis consisted of defining Area of Interest (AOI), which included only the 5 host plasma cells, and then for specific areas in the worm sections (i.e. the hypodermis, etc.). Examples of these custom AOIs are shown in Figs. 1b, 2b and 2d. A mean value of intensity (0–255) was generated from each bitmap to represent the entire hypodermis or all 5 of the host plasma cells. The gray scale values for both the hypodermal areas, and the positive control plasma cells were averaged for each nodule before comparing ivermectin-treated nodules with untreated nodules. An unpaired t-test was used to determine if there was a statistically significant difference between the two groups, ivermectin-treated and untreated nodules, using a p value of ≤ 0.05 as significant and 80 % power to detect the difference.

### Co-localization observations

Analysis for co-localization of WSP and Nras/let-60 staining was accomplished on a Zeiss LSM Pascal (Carl Zeiss Microscopy, Thornwood, NY) using the dyes Alexa Fluor® 488 (green) and 647 (red) (Life Technologies) to detect Nras/let-60 and WSP, respectively. Images were captured using a 63× oil objective at the maximum resolution. Co-localization was assessed using the software accompanying the Zeiss Pascal microscope generating the Manders’ correlation coefficient presented in Table 3 [24]. Co-localisation was further examined by the Costes method [25] using the add-on program JACoP for imageJ [26].

### Statistical analysis

When assessing specific changes in onchocercal nodules, a collection of a number of coiled worms, using histological approaches it is necessary to avoid the error of “repeated measuring” (i.e. avoid pseudo-replication error). To do this we regarded each nodule as an individual entity (sample). Thus with each nodule we assessed 5 worm sections located in the geometric centre of each of three “worm” in each treatment group; a power calculation indicated that this would give a statistically significant comparison between the two treatment groups. An unpaired t-test was used to analyze the gray scale images of the samples in each group. The power to detect was set at 80 %. A *p*-value ≤ 0.05 was considered significant. All calculations were performed in Microsoft Excel® 2007.

## Results

### Nodule status

**Fig. 3:**
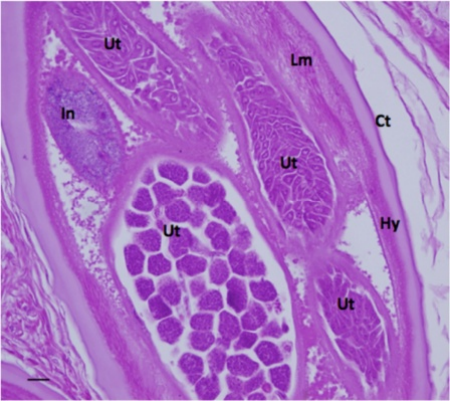
A healthy adult untreated female *O. volvulus* demonstrating multiple developing forms in the uterus (H&E stain). Key: Ct: cuticle, Hy: hypodermis, Lm: longitudinal muscle, In: intestine, Ut: uterus

The main components of adult female *O. volvulus* are shown in Fig. 3. There was no obvious morphological evidence in any of onchocercal nodules used in this study that was suggestive of major degeneration, destruction or death of worms, other than changes normally seen in in aging *O.volvulus* adult worms (such as an accumulation of pigment in the intestine). The vast majority of worms observed in all samples, both treated and untreated, was considered to be viable from a morphological perspective.

### Distribution of Nras staining

**Fig. 4:**
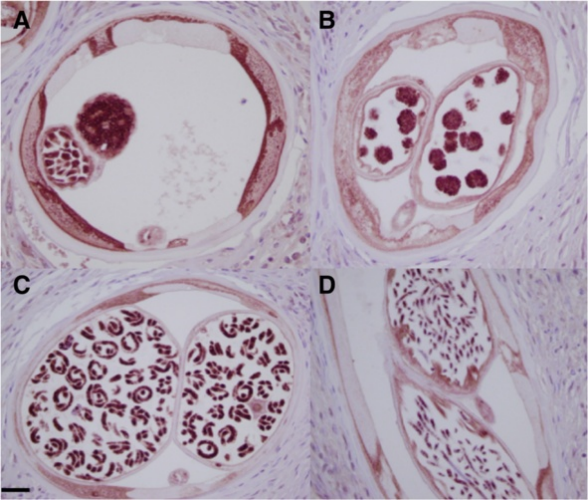
Positive anti-Nras/let-60 staining: **a** Oocytes. **b** Morulae. **c** Coiled microfilariae. **d** Limited staining is seen in stretched microfilariae

Nras/let-60 staining was detected in discrete areas of the parasite and was also pronounced in host plasma cells (Fig. 1), with no confounding background staining. Plasma cells were the predominantly stained host cell with weaker staining present in some macrophages and endothelial cells. In the adult worm, positive staining was seen in the hypodermis and the uterine epithelium, the developing embryonic and germ line forms; staining in other tissues was weaker and less consistent. Staining was also seen in the intestine, most prominently in the epithelium and, with the strongest staining in these epithelial nuclei. Staining was never detected in the longitudinal muscle or cuticle (Fig. 4). The staining in stretched mff was confined to the nuclei. A generally diffuse pattern of staining was also observed in the hypodermis with punctate staining in zones known to be inhabited by *Wolbachia*. As mentioned, the strongest staining was seen in developing forms present in females containing actively dividing cells, such as those present in morulae (Fig. 2 and Table 1). In males, spermatocytes strongly stained while spermatozoa were almost free of positivity (not shown). These patterns were consistent throughout all worm sections and nodules analysed.

### Comparison of Nras staining in untreated and ivermectin-treated *O. volvulus*

Nras/let-60 staining was consistent across all untreated parasites, being most pronounced in areas such as the peri-nuclear zone and the internal border of the hypodermis, as well as the epithelium of the uterus (Fig. 2 and Table 1). Staining was either absent or was remarkably decreased in intensity in ivermectin-treated parasites (Fig. 2c), and was virtually absent from the zones generally inhabited by *Wolbachia*. Nuclei in the tissues that were positive in untreated worms exhibited a marked decrease in staining intensity in the worms from ivermectin-treated patients. When present, developing forms also exhibited decreased stain intensity (Fig. 2c) in this group. Image analysis indicates a significant difference between treated and untreated parasites *p* = 0.0175 (Table 2). No differences were seen between these two groups in staining intensity of host plasma cells (used as stain controls).

**Table 2:** Comparison of staining positivity with anti-Nras/let-60 antisera in untreated and ivermectin treated *Onchocerca volvulus* (seen in Fig. 4). Statistical analysis of grey-scale images^a^

Nodule origin	Sample size (nodules)
Untreated *O. volvulus*	3
Ivermectin treated *O. volvulus*	3
p-level (0.05)	0.0175

^a^t-test values: mean for Untreated = 158.02 with variance = 11.589. Mean for Treated = 167.94 with variance = 7.786

### Identification of *Wolbachia*

**Fig. 5:**
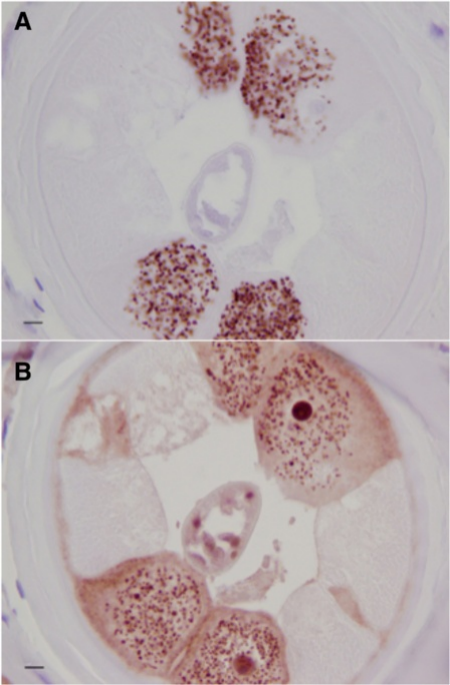
**a** Female *O.volvulus* stained with anti-WSP demonstrating the presence of *Wolbachia*. **b** A serial section stained the anti-Nras stain showing an intense punctate pattern associated with *Wolbachia*

Anti-WSP staining clearly identified individual organisms in various tissues (Fig. 5a); staining intensity and distribution were independent of the ivermectin treatment status. The number of bacteria varied within a tissue, such as the hypodermis, depending on the location within the worm, with some areas being free of organisms, whereas large numbers were present in others. Variation in presence and number of *Wolbachia* in tissues (asymmetrical distribution) was also evident in developing forms. As Nras/let-60 staining also exhibited a punctate nature in certain areas of the worm, namely those reported to contain *Wolbachia,* such as the hypodermis, a BlastP search was undertaken to investigate the possibility that an Nras/let-60 homolog was present in *Wolbachia.* This search compared human Nras and *B. malayi* let-60 to the *Wolbachia* endosymbiont of *B. malayi*. In both cases, the closest bacterial protein returned was Elongation Factor 4 *Wolbachia* (EF-4), previously annotated as GTP-binding protein Lep-A [GenBank YP_198497.1]. A global amino acid alignment showed very low sequence homology between this protein and human Nras or *B. malayi* let-60 (Additional file 2: Figure S2), suggesting that the anti-human Nras antibody is unlikely to recognize EF-4 in *Wolbachia*.

### Co-localisation of Nras/let-60 and WSP

**Fig. 6:**
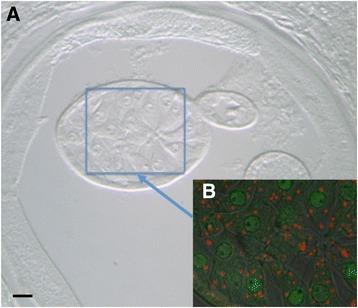
The primary growth zone of a female *O. volvulus.* **a** Differential interference contrast (DIC) image of the area shown in 6B. **b** Laser scanning miscroscope (LSM) image of mature primary oocytes showing virtually no co-localization as demonstrated by the lack of yellow color in the image

**Fig. 7:**
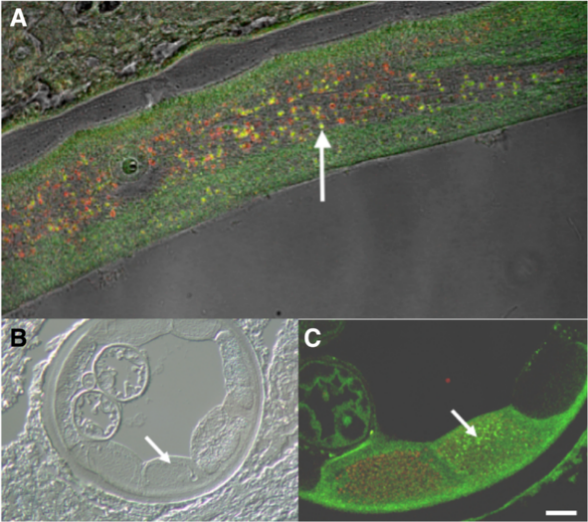
**a** LSM image of the hypodermis of an adult female *O. volvulus* demonstrating co-localization as evidenced by yellow color (Arrow). **b** DIC image of the site shown in 7C. **c** LSM image of the hypodermis of an adult female *O. volvulus* demonstrating co-localization as evidenced by yellow color (Arrow)

Red fluorescence due to presence of red Alexa® Fluor 647 stain, indicating the presence of WSP, was confined to punctate areas in the hypodermis and germ line tissues (Figs. 6 and 7). In the hypodermal sections tested, this stain was co-localized with the green 488 stain for Nras/let-60 (Table 3), and thus was seen as yellow colour in the representative images (Fig. 7). Green (Nras/let-60) was the predominant colour in the hypodermal sections, indicating the presence of more abundant Nras/let-60 staining compared to WSP. Yellow coloured pixels, representing co-localization of Nras/let-60 and WSP, were observed in all hypodermal sections (Table 3); this supports the conclusion that *Wolbachia* is commonly co-localized with Nras/let-60 in *O. volvulus*. The hypodermal sections were analysed further using Manders’ Correlation Coefficient and Costes P-value [24, 25]. This conclusion is also supported by light microscopic analyses, in which structures with punctate Nras/let-60 staining appeared, from serial sections, to reside in the same anatomical location as those detected by anti-WSP (Fig. 5a and b).

**Table 3:** Confocal Analysis. Determination of the overlap between WSP and Nras/let-60 fluorescent staining. Co-localization of anti-Nras staining with anti-WSP staining in two different locations in untreated *Onchocerca volvulus*

Anatomical location	Manders’ correlation coefficient	Costes *P*-value
Hypodermis^a^	0.43	1
Hypodermis^b^	0.305	1

^a^Area studied is shown in Fig. 7a

^b^Area studied is shown in Fig. 7c

### Doxycycline treated nodules

In a small pilot study examining a few doxycycline-treated nodules it was observed (data not shown) that there was the expected reduction in *Wolbachia* induced by the drug, which was paralled by a corresponding reduction in Nras/let-60 staining in the hypodermis. The Nras/let-60 staining in the uterine epithelium appeared unchanged. This supports the contention that there is an intimate relationship between *Wolbachia* and the host worm let-60.

### Co-localization in other filarial species

**Fig. 8:**
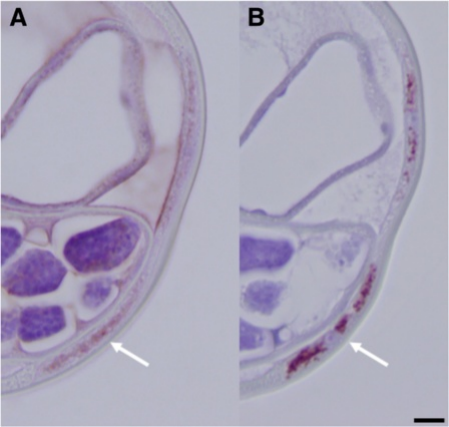
Adult female *Brugia malayi.* **a** Anti-Nras stained section showing a punctate staining present in the hypodermis. **b** Anti-WSP stain of a serial section showing punctate staining associated with the presence of *Wolbachia*

**Fig. 9:**
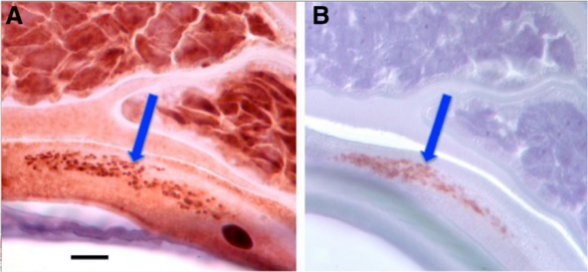
Adult female *Dirofilaria immitis.* **a** Anti-Nras stained section showing punctate staining associated with the *Wolbachia*. **b** An anti-WSP stained serial section

**Fig. 10:**
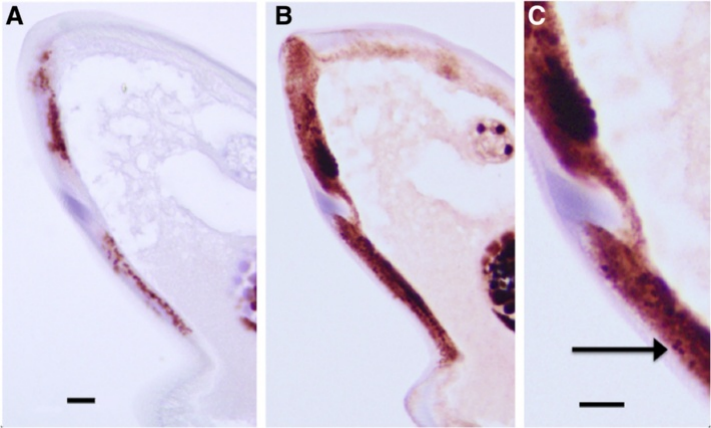
Adult female *L. sigmodontis*. **a** An anti-WSP stained section showing the presence of *Wolbachia*. **b** An anti-Nras stained serial section of 10A. **c** A higher magnification of 10B showing the punctate staining associated with the presence of *Wolbachia*

**Fig. 11:**
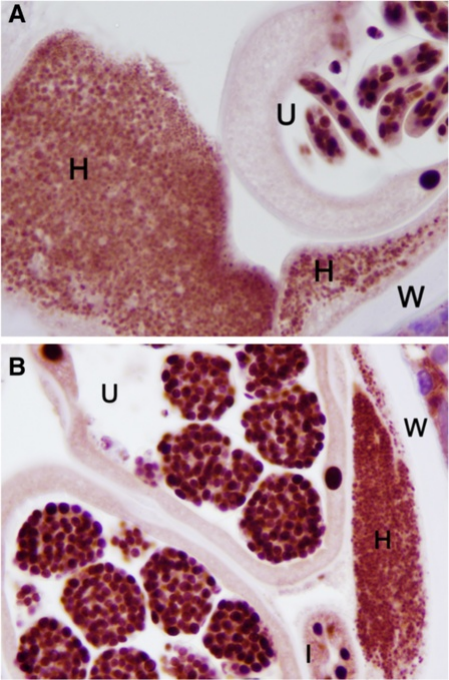
Adult *Onchocerca ochengi* stained with anti-Nras. **a** The hypodermis (H) showing punctate staining consistent with the location of *Wolbachia*. Nuclei in the uterine (U) developing microfilariae are also positive. **b** The nuclei in the developing morulae in the uterus (U), and the intestinal epithelium (I) are also positive. The body wall (W) cuticle is negative

Specimens of *L. sigmodontis*, *B. malayi* and *D. immitis*, *O.ochengi*, all known to harbor *Wolbachia,* showed a similar picture of Nras/let-60 and WSP co-localization (Figs. 8, 9 and 10). The *Nras positivity* in *Onchocerca ochengi* (Fig. 11) was seen to be in a similar location (the hypodermis) as other filariae and where *Wolbachia* are likely to be present although we did not carry out WSP staining on this nematode in this present study.

## Discussion

This present study has identified that Nras/let-60 is present in *O. volvulus* and can be found in many tissues within this parasite (Table 1), especially those that are involved in active replication, as might be expected. As it is also significantly present in the hypodermal syncytial cells, cells that are not undergoing replication but supporting the cuticle, maintaining ionic integrity and the nervous system, it is possible that Nras/let-60 is also involved with general homeostasis of the worms. We suggest therefore that this protein is probably of importance to the general well-being and longevity of the worm. The lack of staining in muscle, and the decrease in staining in mature mff that are ready to be released, may reflect a reduced need for this protein in these particular tissues.

A direct relationship between Nras/let-60 and parasite longevity is a logical conclusion from our data presented here derived from the comparison of untreated worms with ivermectin-treated parasites. Our observations of reduced Nras/let-60 staining after prolonged ivermectin treatment suggest that this drug has a significant effect on Nras/let-60 expression in tissues of *O. volvulus*. How ivermectin affects adult *O. volvulus* still remains unclear, but it is believed that the drug paralyses the uterus, preventing mff release. In addition, it has been reported that repeated ivermectin treatments induce a slow loss of viability and reproductive ability in *O. volvulus* [5, 8]; The observations we make here support the hypothesis that there may also be a general debilitating effect of ivermectin on adult *O. volvulus*, which may result in a reduction in longevity and/or reproductive competence in these parasites. Our findings suggest a possible effect of ivermectin on the activity and biochemical integrity of *Wolbachia* via Nras systems that may be involved in the described lack of significant macrofilariacidal activity of doxycycline in *Dirofilaria* infections unless it is used with ivermectin [27, 28]. In this latter nematode species the enhancement of the anti-*Wolbachia* properties of doxycycline by ivermectin may be due to ivermectin’s effect on the production or maintenance of Nras.

The parasitological observation of a diminished number of early larval stages in ivermectin-treated worms (Table 1) may reflect the down-regulation of Nras/let-60 expression, perhaps through the interruption of mitosis. To support this hypothesis a basic pathological examination was undertaken initially to assess the health of the parasites present independently of Nras/let-60 staining. There was no morphological evidence in the worms that would support a significant difference in nematode health due to aging or other damaging processes [29]. It is possible that age of worms could be a confounding factor in the observations we have made. However, although this possibility exists, the uniformity of staining seen throughout the control samples (which are likely to contain worms of different ages) suggests that there was not a major effect due to age on Nras/let60 staining in adult worms.

An additional finding in this study is the close morphological association between Nras/let-60 and *Wolbachia* in *O. volvulus*; a finding which was extended to the 3 additional filarial species suggesting that this is a common phenomenon in filariae. The punctate Nras/let-60 staining in distinct areas of *O. volvulus* adult females where *Wolbachia* are known to reside suggests a distinct relationship between this protein and the endosymbiont; although determining whether Nras is actually a physical component of the bacteria, or simply very closely associated but still outside the actual endosymbiont, cannot be achieved conclusively in a light microscopical study. Our data also showed that this morphological association of *Wolbachia* with Nras/let-60 is not universal, as is seen in the case in the primary growth region (Fig. 6) where many *Wolbachia* are free of an association with Nras/let-60. This could be explained by a differential biochemical activity of *Wolbachia* organisms in the different locations within the worm. Such a difference might be related to the stage of development of both the Wolbachia and/or the parent worm itself. Previous studies have shown that *Wolbachia* divide rapidly in the syncytium of the oogonia prior to cellularisation [14]. It has also been shown that *Wolbachia* has the ability to act as a secondary mitochondrion [30] and this could explain the co-localization observed in the hypodermis of the adult filariae where *Wolbachia* are thought to slowly divide [31].

BLASTp analysis indicated that *Wolbachia* only possess the GTP binding protein EF-4: suggesting that *Wolbachia* may be utilizing the host’s let-60. This conclusion, however, depends on the functions that let-60 performs at the *Wolbachia*–host interface, a subject that has received no attention. However, we were unable to determine if the observed staining is within the bacterium or at the interface between host and symbiont. It is possible that it is recruited to the interface to serve the needs of the host, independent of the symbiont. Given the high degree of sequence homology between the nematode let-60 and human Nras, and that *Wolbachia* lacks a similar homolog, we propose that let-60 may perform the same function in *Wolbachia* as it does in the nematode. Further evidence of a mutualistic relationship is shown by the lack of co-localization in primary oocytes (Fig. 6b) where the red signal is not co-localized with the weaker green signal. This suggests again that quiescent *Wolbachia* not undergoing division may not require let-60, in which case the host worm does not need to provide it; there is, of course, the caveat that we cannot determine in this present study whether the staining is within the symbiont or outside of it. This differential biochemical activity could be due to cellular division of the *Wolbachia*, which exist in a homeostatic relationship with the worm [32].

The only drug presently capable of safely killing *O. volvulus* adults, doxycycline, has a direct effect on *Wolbachia* [11, 12]. Our observation that *Wolbachia* are associated with Nras/let-60 raises the question as to whether the reduction in Nras/let-60 presence adversely affects *Wolbachia* and consequently contributes to the slow degeneration of the adult. Ivermectin treatment appears to result in a reduction in Nras/let-60 in the parasite and in its association with *Wolbachia*. It is clearly not the destruction of Wolbachia by ivermectin that contributes to the loss of viability of the host worm during treatment, but rather it could be the effect ivermectin has on metabolic processes including those propelled by Nras/let-60. Studies to validate Nras/let-60 as a biomarker of nematode viability when exposed to macrofilaricides have begun using agents such as flubendazole [3]. Studies using this marker on worms exposed to the anti-*Wolbachia* agent doxycycline will also be informative [33].

Validated biomarkers are needed not only to develop a better understanding of the antifilarial pharmacology of ivermectin and other anthelminthics, but also in general for assessing adult filarial health. Such biomarkers are urgently needed to speed the development of new macrofilaricidal agents [1, 4]; similarly, biomarkers of viability are needed to guide end-stage campaigns for control programmes. Current methods include estimates of motility (visual or automated) [34, 35] and metabolic competence using the (3-(4, 5-dimethylthiazol-2-yl)-2, 5-diphenyltetrazolium bromide (the MTT assay [36])). However, these methods may not reveal subtle damage that eventually leads to parasite death, and are difficult to apply to adult filariae in tissues or nodules. Simple numerical analyses of patterns and intensity of histo-chemical staining with commercially available antibodies are likely to be highly valuable for these purposes and could lead to more standardization among the different laboratories involved in drug trials. Whereas most current pathological descriptions of viability in studying anthelmintic activity rely on subjective analysis of sections with agreement from multiple parties [21], the system developed in this present study allows for objective comparison of staining intensity and distribution among multiple specimens.

The numerical analysis we have carried out in this present study reflects and supports the difference seen when directly observing the staining intensity between these two groups under a microscope. Although the numerical difference in this study is admittedly small (i.e. 6 %) although statistically significant, it should be said that the parasite-drug system (ivermectin use in MDA programmes) might show such a low numerical value due to relatively minor effects of this particular drug on the adult worms. However, it is quite possible that such changes (reduction in Nras presence) may be much more dramatic in worms subjected to more damaging agents. It is our belief that the subjective scores currently used to assess adult worm damage are extremely vague and subject to a great deal of observer variation, and that an additional marker such as Nras will improve the definition of worm damage. Further usage in different model systems will reveal if this particular marker is a major advance. We feel that the use of such morphological approaches that have a strong molecular background (exampled in our data here with the discussion on the relationship between this particular marker and a major component such as Wolbachia) contributes to a more accurate definition of damage and degeneration and to a better fundamental understanding of the currently very vaguely characterised phenomenon of the gradual demise of a nematode *in situ*.

Thus, we propose that Nras/let-60 be added to the very limited arsenal of biomarkers presently available to characterize worm health [21, 36–38]. This highly conserved protein is involved in germ line morphogenesis, vulval development, excretory tube development and overall development and ageing. The closest well-characterized Ras homolog from nematodes, let-60 from *C. elegans* [16, 17], has close homologs in genomes of filariae, including *B. malayi, O. volvulus* and *L. loa* [10, 23]. The presence in *L. loa*, a nematode without *Wolbachia* suggests the integral nature of let-60 to the worm and not merely its presence associated with *Wolbachia*. Using genomic sequence comparisons across multiple species may be a useful method for identifying additional biomarkers for parasite viability.

## Conclusions

A histological approach using an anti-Nras antibody supports the conclusion that this protein is a candidate biomarker for viability and health of *O. volvulus*, and most likely other filariae, as it can distinguish between ivermectin-treated and untreated adult *O. volvulus*. In addition, we find that Nras/let-60 significantly co-localizes with the endosymbiont *Wolbachia* in *O. volvulus* and other filariae.

## Additional files

Additional file 1: Figure S1. Global protein alignment between *B. malayi* [GenBank: XP_001899045.1] and human [GenBank: NP_002515]. Global protein alignment between *L. loa* [Genbank XP_003139513.1] and human [GenBank: NP_002515]. Additional file 2: Figure S2. Global protein alignment between *Brugia malayi* let-60 [GenBank: XP_001899045.1] and Elongation Factor 4 *Wolbachia*, previously annotated as GTP-binding protein Lep-A [GenBank: WP_011256865.1].
