# Supplementary material for: A histochemical study of the Nras/let-60 activity in filarial nematodes
Source: Parasit Vectors. 2015 Jul 1;8:353. doi: 10.1186/s13071-015-0947-6 (PMC4493820; doi:10.1186/s13071-015-0947-6)
Supplement: Additional file 1: Figure S1. — Global protein alignment between B. malayi [GenBank: XP_001899045.1] and human [GenBank: NP_002515]. Global protein alignment between L. loa [Genbank XP_003139513.1] and human [GenBank: NP_002515]. [file 13071_2015_947_MOESM1_ESM.docx]

XP_001899045. 1 MTEYKLVVVGDGGVGKSALTIQLIQNHFVEEYDPTIEDSYRKQVVIDGET 50

||||||||||.||||||||||||||||||:||||||||||||||||||||

NP_002515.1 1 MTEYKLVVVGAGGVGKSALTIQLIQNHFVDEYDPTIEDSYRKQVVIDGET 50

XP_001899045. 51 CLLDILDTAGQEEYSAMRDQYMRTGEGFLLVFAVNEAKSFENVTQYRDQI 100

|||||||||||||||||||||||||||||.|||:|.:|||.::..||:||

NP_002515.1 51 CLLDILDTAGQEEYSAMRDQYMRTGEGFLCVFAINNSKSFADINLYREQI 100

XP_001899045. 101 RRVKDSDEVPMVLVGNKCDLAQRTVESRAILDASRSLGMPAVETSAKTRM 150

:||||||:||||||||||||..|||:::...:.::|.|:|.:|||||||.

NP_002515.1 101 KRVKDSDDVPMVLVGNKCDLPTRTVDTKQAHELAKSYGIPFIETSAKTRQ 150

XP_001899045. 151 GVDDAFYTLVREIRKHKEKQ----------CIKPRKKRKCVII 183

||:|||||||||||:::.|: |: ...||::

NP_002515.1 151 GVEDAFYTLVREIRQYRMKKLNSSDDGTQGCM----GLPCVVM 189

XP_003139513. 1 MTEYKLVVVGDGGVGKSALTIQLIQNHFVEEYDPTIEDSYRKQVVIDGET 50

||||||||||.||||||||||||||||||:||||||||||||||||||||

NP_002515.1 1 MTEYKLVVVGAGGVGKSALTIQLIQNHFVDEYDPTIEDSYRKQVVIDGET 50

XP_003139513. 51 CLLDILDTAGQEEYSAMRDQYMRTGEGFLLVFAVNEAKSFENVTQYRDQI 100

|||||||||||||||||||||||||||||.|||:|.:|||.::..||:||

NP_002515.1 51 CLLDILDTAGQEEYSAMRDQYMRTGEGFLCVFAINNSKSFADINLYREQI 100

XP_003139513. 101 RRVKDSDEVPMVLVGNKCDLAQRTVESRAILDASRSLGMPAVETSAKTRM 150

:||||||:||||||||||||..|||:::...:.::|.|:|.:|||||||.

NP_002515.1 101 KRVKDSDDVPMVLVGNKCDLPTRTVDTKQAHELAKSYGIPFIETSAKTRQ 150

XP_003139513. 151 GVDDAFYTLVREIRKHKEKQ----------CVKPRKKRKCVII 183

||:|||||||||||:::.|: |: ...||::

NP_002515.1 151 GVEDAFYTLVREIRQYRMKKLNSSDDGTQGCM----GLPCVVM 189

Additional file 1: Figure S1. Global protein alignment between *B. malayi* [GenBank: XP_001899045.1] and human [GenBank: NP_002515]. Global protein alignment between *L. loa* [Genbank XP_003139513.1] and human [GenBank: NP_002515].
