# Supplementary material for: A histochemical study of the Nras/let-60 activity in filarial nematodes
Source: Parasit Vectors. 2015 Jul 1;8:353. doi: 10.1186/s13071-015-0947-6 (PMC4493820; doi:10.1186/s13071-015-0947-6)
Supplement: Additional file 2: Figure S2. — Global protein alignment between Brugia malayi let-60 [GenBank: XP_001899045.1] and Elongation Factor 4 Wolbachia, previously annotated as GTP-binding protein Lep-A [GenBank: WP_011256865.1]. [file 13071_2015_947_MOESM2_ESM.docx]

Top of Form

Bottom of Form

XP_001899045. 1 ---MTEYKLVVVGDGGVGKSALTIQLIQ-----------NHFVEEYD--- 33

:..:.::...|. |||.|..:||: |..::..|

WP_011256865. 1 MDNVRNFAIIAHIDH--GKSTLADRLIEECNGLEAREMTNQVLDSMDIER 48

XP_001899045. 34 --------PTIEDSYRKQVVIDGETCLLDILDTAGQEEYSAMRDQYMRTG 75

.|:..:|... ||....|:::||.|..::|....:.:...

WP_011256865. 49 ERGITIKAQTVRLNYTAN---DGNRYCLNLMDTPGHVDFSYEVSRSLAAC 95

XP_001899045. 76 EGFLLVFAVN---EAKSFENVTQYRDQIRRVKDSDEVPMVLVGNKCDLAQ 122

||.|||...: ||::..|| .:.:.::.|:.:|| ||.||..

WP_011256865. 96 EGSLLVIDSSQGVEAQTLANV------YKAIDNNHEIIVVL--NKVDLPA 137

XP_001899045. 123 RTVESRAILDASRSLGMPAVET---SAKTRMGVDDAFYTLVREIRKHKEK 169

...| |..|.....:|:.:.|: ||||.:|:.|....::.::

WP_011256865. 138 SDPE-RVKLQVEEIIGIGSSESILISAKTGLGIRDVLEAIITKL------ 180

XP_001899045. 170 QCIKPRKK------RKCVII------------------------------ 183

|..: .:.:::

WP_011256865. 181 ----PAPQGNINAPLQAILVDSWYDPYLGVVILVRVKNGVLKKGMKIVMM 226

XP_001899045. 184 -------------------------------------------------- 183

WP_011256865. 227 SNNAVYQVDNIGIFTPKKVMTSELSAGEVGFITASMRKMADCKVGDTITE 276

XP_001899045. 184 -------------------------------------------------- 183

WP_011256865. 277 EKKPCGEALPGFKEIHPVVFCSIFPNKTDGFKYLREALEKLHLNDTSFTF 326

XP_001899045. 184 -------------------------------------------------- 183

WP_011256865. 327 EAETSNALGYGFRCGFLGMLHLEVIQERLEREFDLDLTATAPSVIYEVTT 376

XP_001899045. 184 -------------------------------------------------- 183

WP_011256865. 377 QSGETLNIHNPNDMPDSVKIKIVEEPWITATIMVPDQYLGEILSLCDERR 426

XP_001899045. 184 -------------------------------------------------- 183

WP_011256865. 427 GKQEDLSYVGNTMTALLKYKLPLSEVVLDFYDRLKSISKGYASLDWEISN 476

XP_001899045. 184 -------------------------------------------------- 183

WP_011256865. 477 YQESQIDKLSFLVNGEPVDALACIVHKSRSEKRGREICARLRDLIPRQQY 526

XP_001899045. 184 -------------------------------------------------- 183

WP_011256865. 527 KIAIQATVGGKIVARETINPYRKDVTAKLYGGDVTRKMKLLEKQKKGKKR 576

XP_001899045. 184 ---------------------- 183

WP_011256865. 577 LYSVGNVDIPHNAFIQALKIGD 598

Additional file 2: Figure S2. Global protein alignment between *Brugia malayi* let-60 [GenBank: XP_001899045.1]and Elongation Factor 4 *Wolbachia*, previously annotated as GTP-binding protein Lep-A [GenBank: WP_011256865.1].
